# Crop Pest Identification and Real-Time Monitoring System Design Based on Improved YOLOv8s

**DOI:** 10.3390/s26020404

**Published:** 2026-01-08

**Authors:** Qiang Gao, Chongchong Shi, Yu Ji, Meili Wang

**Affiliations:** 1 School of Information Engineering, Xi’an University, Xi’an 710065, China; 2 Shaanxi Joint Laboratory of Artificial Intelligence, Xi’an University, Xi’an 710065, China; 3 School of Computer Science, Xijing University, Xi’an 710000, China; 4 College of Information Engineering, Northwest A&F University, Yangling 712100, China

**Keywords:** crop pest detection, improved YOLOv8s model, system design, lightweight attention mechanism, feature enhancement module

## Abstract

This study aims to address the limitations of the YOLOv8 model in terms of low detection accuracy and poor deployment adaptability in the context of crop pest detection. To this end, a lightweight attention mechanism and a feature enhancement module were incorporated into the structure of YOLOv8s, with a view to optimizing its detection performance across a range of insects. Based on this improved model, a real-time pest monitoring system was further developed. Results showed that on the self-constructed pest dataset, the proposed improved model increased mAP_0.5_, and mAP_0.5–0.95_ by 0.6% and 0.8%, respectively, and reducing the number of model parameters from 11.1 × 10^6^ to 10.2 × 10^6^ compared to the original YOLOv8s model. Using an A40 graphics card at 640 × 640 resolution with a batch size of 32, the inference speed reached 249.76 frames per second, representing a modest improvement over the original model’s 225.38 frames per second. On the IP102 dataset, the proposed improved model increased Precision (P), mAP_0.5_ and mAP_0.5–0.95_ by 2.6%, 2.7% and 1.4%, respectively, compared to the original YOLOv8s model. This study demonstrated that the proposed model exhibited a high level of recognition accuracy for pests in different states, thereby providing a valuable reference for the accurate identification of crop pests.

## 1. Introduction

With the advancement of modern agriculture, pests pose an increasingly significant threat to crop yield and quality. Conventional manual detection of crop pests presents several limitations, which considerably hinder the timeliness and efficacy of pest monitoring [1]. Regarding detection efficiency, manual methods depend entirely on field-by-field and plant-by-plant inspections conducted by personnel. Even experienced agronomists can only inspect a limited area of farmland within a single day [2]. For large-scale planting bases, significant manpower and time inputs are often required; furthermore, it remains challenging to achieve the rapid census and dynamic monitoring of the pest infestations, and the optimal window for pest control is easily missed. In terms of detection accuracy, manual assessment relies heavily on individual experience [3]. On the one hand, for pests with small sizes and body colors highly similar to those of crop leaves or soil, accurate identification and counting via the naked eye are challenging, often resulting in missed detections or misclassifications. On the other hand, pests exhibit morphological differences at various growth stages, and some pests are similar in appearance to beneficial insects. Accurate differentiation of these pests requires professionally trained personnel, in the absence of such expertise, pest surveillance data tends to be inaccurate, which further impairs the scientific formulation of subsequent control strategies.

In recent years, to address the limitations of traditional detection methods, target detection-based intelligent recognition technology for crop pests and diseases has emerged as an important research direction in precision agriculture. Among these, the YOLO series models have been extensively utilized in agricultural pest identification, due to their end-to-end architecture, rapid detection speed, and high accuracy [4]. However, practical field pest monitoring faces dual challenges: on the one hand, the complex natural environment, including leaf shading, soil reflection, and light fluctuations, results in scattered pest distribution and difficult target capture; on the other hand, when pests are collected through trapping equipment (a common engineering solution for field monitoring), new pain points such as high population density, mutual overlap, and species morphological similarity arise [5]. These issues collectively limit the detection accuracy and deployment adaptability of the original YOLOv8 model. To address this problem, researchers have proposed a range of targeted improvement schemes. For example, in the cotton field scenario, Sun et al. proposed the YOLO-UP model [6]. Specifically, by designing the SC3 module to enhance low-level feature extraction and combining the GeLU activation function, AFPN neck network, and large-kernel separable convolution to optimize feature processing, the model achieves efficient detection of high-density small-target pests. Notably, the mAP_0.5_ of the model is 3.46% higher than that of the YOLOv8n model. In citrus pest and disease detection, Luo et al. proposed the Light-SA YOLOv8 model [7], which introduced both the BRA self-attention module and the Fast_C2f structure. While maintaining its lightweight nature, the model improves the discrimination of pests and diseases against leaf backgrounds, and mAP0.5 reaches 92.5%. To achieve simultaneous monitoring of multiple crop pests, Vilar-Andreu et al. developed a universal insect detection framework across crops [8]. The model’s generalization performance was significantly improved by combining large-scale data augmentation and category integration, with an average mAP_0.5_ of 0.967. In research on detection in specific crop scenarios, Zhang et al. proposed the RSD-YOLOv8 model framework [9]. By combining the RepLightConv module with the Dyhead module and adopting LAMP pruning, the framework achieved the lightweight design of the forestry pest detection model. The results showed that the mAP_0.5:0.95_ of the model reaches 88.6%. Bruno Lima et al. proposed an improved YOLOv8n model that combines the P2 feature layer and the C2f2 module for detecting of brown stink bugs in soybean fields [10]. This model effectively enhances the capabilities of small-target recognition and real-time tracking. Wang Xingwang et al. combined the Swin Transformer with distributed shift convolution and proposed the YOLOv8-STSF model, which achieved high-precision and real-time mobile-terminal monitoring of multiple types of rice pests [11]. Zhu et al. [12] proposed the Poly-YOLOv8 model, which replaced the rectangular box annotation with a polygonal detection head. The results showed that this model significantly improved the fine localization of pest-infested areas on maize leaves. Sun et al. [13] proposed a lightweight tobacco pest detection model by introducing the VoV-GSCSP and SimAM attention mechanisms, which improved the detection accuracy while reducing the model parameters. Researchers have also explored various optimization strategies to address issues such as tiny pests and complex backgrounds. For example, Wang et al. [14] integrated the SIoU loss function with the BiFormer attention mechanism to achieve high-precision detection of tiny pests in tea gardens, with an accuracy of 98.16%, which is superior to models such as YOLOv9 and YOLOv10. Zhang et al. [15] incorporated the DCF module with the CBM activation function, which effectively mitigated the gradient vanishing during the training on the IP102 dataset and significantly enhanced the model’s robustness. Liu et al. [16] proposed the RicePest-YOLO structure and integrated the ODConv, BiFPN and LAMP pruning to realize model lightweighting; this approach maintained high accuracy in rice pest detection while reducing the number of parameters by nearly half. In addition, some scholars have explored the effectiveness of fusing generative adversarial networks (GANs) with multimodal feature enhancement in pest detection. For example, Liu et al. [17] utilized the StyleGAN2-ADA model to augment corn pest samples, and by integrating FasterNet with the NAM attention mechanism, proposed the FNW-YOLOv8 model, which achieved efficient detection of corn pests. Zheng et al. [18] proposed the Rice-YOLO model, which achieved a balance between detection accuracy and inference speed in rice pest detection through a lightweight detection head and a dynamic sampling module. Xie et al. [19] optimized the detection of pests and diseases on cucumber leaves by integrating DCNv2 and SEAM attention mechanisms; the results showed that the mAP_0.5_ was improved to 75.1%, while the inference speed reached 400 fps. Priya et al. [20] developed a farmer-oriented pest detection and control system based on the YOLOv8 model. Integrated with Web deployment, the system delivers real-time early warning and control guidance for agricultural production.

Based on the above research, it is evident that numerous studies have been conducted on crop pest identification. However, the YOLOv8s model still exhibits limitations in practical pest detection, such as insufficient accuracy and poor recognition performance for small targets. Based on this, this study incorporates a lightweight attention mechanism and a feature enhancement module to optimize the structure of YOLOv8s, proposing the FusedGM-YOLOv8 model to address the pain points of high density and mutual overlap in pest detection.

## 2. Materials and Methods

### 2.1. Dataset

**Figure 1:**
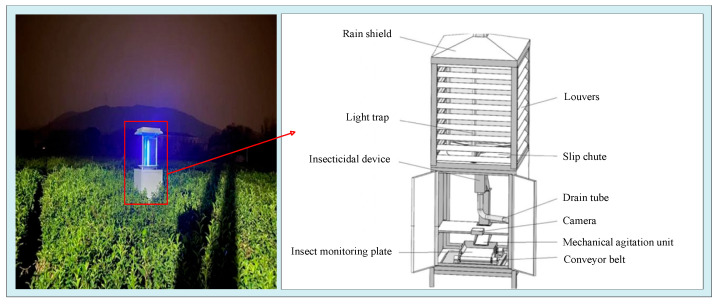
Insect monitoring equipment.

To balance environmental authenticity and image clarity, some studies trap pests using pest monitoring equipment before photographing them [21,22]. This method can separate pests from complex field backgrounds, avoiding interference from the background and lighting on image quality. Moreover, the trapped pests are from actual fields, which can reflect the actual pest species, and the operation requires only placing the equipment in the field. This study deployed intelligent pest image acquisition equipment at crop planting sites and constructed a data acquisition system. To obtain accurate experimental data, this study deployed intelligent pest image-acquisition equipment in the 100 mu core crop planting experimental area and constructed a standardized data-acquisition system. Using a stainless steel frame as the main support and a uniform grid deployment mode, a total of 20 monitoring devices (1 for every 5 mu) are deployed. The equipment is located in the open area between ridges in the crop planting area, avoiding occlusion by tall plants and buildings. The single piece of equipment is fixed to a stainless steel frame by a buried anchor, with the bottom of the frame 50 cm above the ground to ensure stability and accommodate the traffic demand of agricultural machinery in the field. With a stainless steel frame as its main support, the equipment integrates a 20 cm × 25 cm insect-attracting board, using a 500 W HD camera, 12 mm fixed-focus lens with a 30 cm installation height, 5000 K fill light, 20 W ultraviolet light, and a collaborative setup powered by solar energy or 220 V AC (Figure 1). Based on the principle of pest phototaxis and color preference, the insect-attracting light lures pests to stay on the insect-attracting board. Then, a camera with 800 × 600 resolution and JPG image format was triggered at regular intervals to capture pest images on the board. These images were transmitted to the computer terminal in real time via the network for storage and processing. The data collection period spanned from 6 June to 18 July 2025. During this period, the average daily temperature was high, and the insect-attracting boards captured nine types of pests, including bollworm, meadow borer, gryllotalpa orientalis, little gecko, nematode trench, athetis lepigone, armyworm, anomala corpulenta and holotrichia parallela. The trapped pest species were comprehensive and sufficient in quantity, providing high-quality and multi-scenario pest image samples for model training and validation.

The pest monitoring equipment enables efficient collection of field pests by integrating trapping, extermination, and imaging processes. Its operating mechanism and application advantages are as follows: when the equipment is in operation, the ultraviolet insect-attracting lamp emits light at specific wavelengths that are highly attractive to pests, luring them to take the initiative to fly and collide with the equipment’s glass screen. After impact, the pests slide along the funnel structure at the lower end through the open insect-dropping channel into the insecticidal chamber. This chamber adopts far-infrared heating technology to treat the pests: the kill rate of live pests can reach over 90% within 3–5 min after they enter the chamber, and the remaining few survivors will lose mobility or move slowly due to the high temperatures. Subsequently, the immobile pests are transferred to the insect-receiving plate via the electrically controlled flipping base plate, and the high-definition camera on the equipment captures pest images. Since most pests are active at night, the device uses a trap light to capture insects, with the specific time set from 18:00 to 6:00 the next day. The device is equipped with a rain-shielding plate, enabling all-weather insect trapping and killing. The device will collect the pest after killing every 3 h, and then the HD camera will shoot and complete the remote transmission.

**Figure 2:**
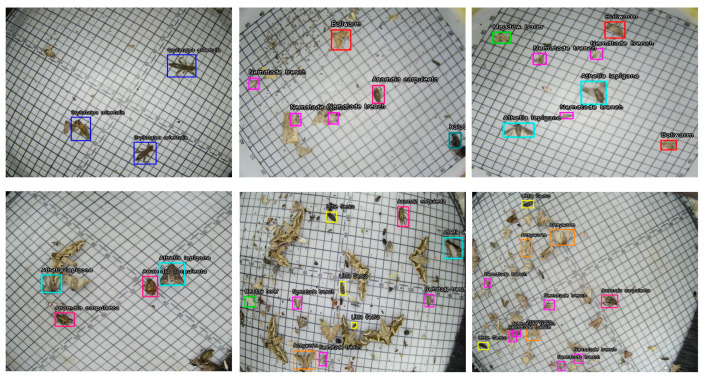
Partial pest samples.

Following image acquisition, pest images of different input sizes were first cropped and unified to a resolution of 640 × 640 pixels, part pest images are shown in Figure 2. Subsequently, the LabelImg annotation tool was used to annotate the leaf disease images, framing the lesion location of each pest, and the lesion locations were saved in. txt format using the YOLO annotation format. Finally, the dataset was divided into training, validation set, and test sets in an 8:1:1 ratio, resulting in 5680 images for training, 710 images for validation, and 711 images for testing. The specific number of labels for each pest is shown in Table 1.

### 2.2. Small Target Detection Algorithm for Pests Based on Improved YOLOv8

YOLOv8 is an algorithm with outstanding performance in object detection [23], which has significant advantages such as rapid detection speed and robust model generalization, and can efficiently address the core requirements of rapid pest detection tasks. Considering the functional requirements of different scenarios and the performance differences in hardware devices in practical applications, the YOLOv8 algorithm has five versions with different specifications (n, s, m, l, and x) designed to achieve flexible matching between scenarios and hardware. From the perspective of model characteristic laws, various versions of YOLOv8 show a clear correlation between performance and resource consumption: versions with a larger number of parameters usually have higher pest detection accuracy, but this is accompanied by a significant simultaneous increase in computational load, which can negatively impact operational efficiency during deployment. To balance detection performance and operational efficiency, and to avoid insufficient detection accuracy that may result from an excessively small number of parameters (e.g., the n version) and circumventing the sharp surge in computational load caused by an excessively large number of parameters (e.g., the m, l, and x versions), this study selects the YOLOv8s version for rapid pest detection.

The YOLOv8 network structure is divided into four core modules: the input end, backbone network, neck network, and detection head. Compared with YOLOv5, YOLOv8 has achieved targeted improvements in all four modules of its core architecture [24], resulting in significant performance improvements: the input end abandons the fixed Mosaic data augmentation of YOLOv5 and adopts an augmentation strategy combining dynamic Mosaic, MixUp, and CutMix. This not only avoids excessive cropping of small targets but also enhances the model’s adaptability to complex scenarios, effectively reducing the risk of overfitting during training. The backbone network replaces the C3 module of YOLOv5 with the C2f module. By adding branch structures and more gradient flow paths, it enhances feature extraction capability without significantly reducing computational efficiency—especially for more accurately capturing detailed features of medium- and low-resolution pests and small targets. The neck network retains the PAN structure but uniformly replaces the original C3 module with the C2f module. Meanwhile, it optimizes the channel weight distribution in feature fusion, making the fusion of features at different scales (e.g., pest contour features) more efficient. This solves the problem of information loss during the transmission of some scale-specific features in YOLOv5 and improves the consistency of multi-scale target detection. In terms of the detection head, the Anchor-Based anchor box detection mechanism of YOLOv5 is abandoned, and an Anchor-Free design (without anchor boxes) is adopted. By directly predicting the target center point and aspect ratio, this approach reduces the manual parameter-tuning cost of anchor box presetting but also avoids problems such as missed or false detections caused by anchor box mismatch.

**Figure 3:**
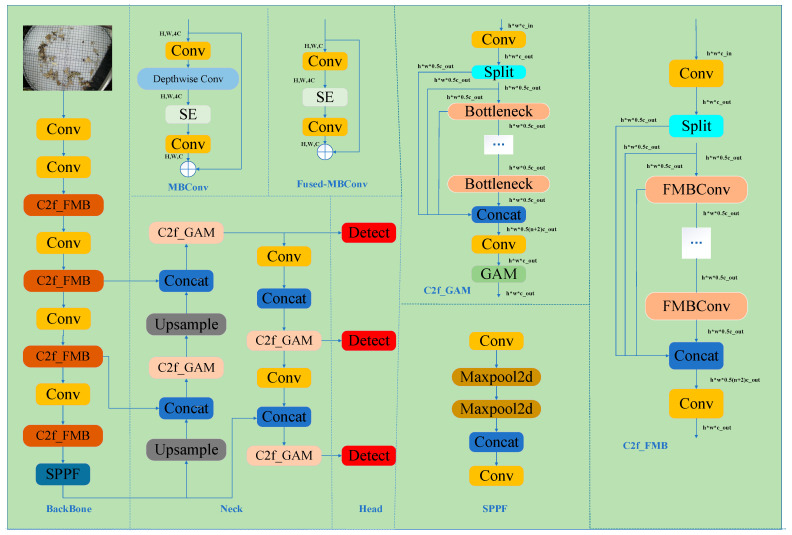
Model architecture diagram of FusedGM_YOLOv8. (Conv is the convolution operation; SPPF is the spatial pyramid pooling module; C2f_FMB is a C2f improved module that integrates multi-scale bottlenecks, while C2f_GAM is a C2f module that incorporates global attention; Head is the detection head; Concat is the feature connection module).

YOLOv8 still has deficiencies in small target detection and feature extraction under complex backgrounds, which are mainly manifested in its limited ability to capture fine-grained features and in interference with target-backgrounds distinction. To address this issue, this study focuses on enhancing the fine-grained feature modeling capability in the backbone network: on the one hand, by introducing the concepts of depth-wise separable convolution and feature dimension expansion into the convolutional structure, the feature expression capability is effectively enhanced while maintaining the network’s lightweight property, thereby better adapting to small target detection scenarios; on the other hand, by integrating the channel attention mechanism to achieve adaptive enhancement of key information, the model can focus on features that are more critical to the detection task. Meanwhile, in the feature fusion stage of the neck network, given YOLOv8’s insufficient ability to model global spatial relationships, this study enhances the network’s focusing capability on salient target regions and effectively suppresses background noise interference by combining a channel attention mechanism with a large convolutional kernel-based spatial modeling strategy. Overall, these improvements have specifically alleviated the shortcomings of YOLOv8 in small target detection and complex scenarios, achieving comprehensive enhancements in the network’s feature discrimination and detection accuracy. The structure of the FusedGM-YOLOv8 algorithm for small pest targets proposed in this study is shown in Figure 3.

### 2.3. Improved C2f_GAM Module

**Figure 4:**
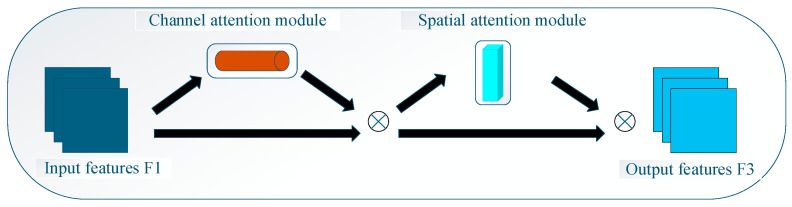
Schematic diagram of the GAM.

To enhance the performance of YOLOv8 in pest detection, this study proposes to incorporate the Global Attention Mechanism (GAM) [25] as an enhancement module. To address the complex background interference in pest images, GAM (Figure 4) enhances the model’s detection capability by strengthening key information, suppressing redundant backgrounds, and designing a module for accurately extracting the salient features of pests. Meanwhile, this study innovatively introduces a dynamic non-monotonic focusing mechanism. Based on the concept of “outliers”, this mechanism evaluates the quality of anchor boxes, enabling the detector to optimize by integrating anchor boxes of various qualities. This further adapts to the detection requirements across different scenarios, thereby improving the accuracy of the results. In pest datasets with multiple background interferences (e.g., crop branches, leaves, and soil), GAM has significant advantages over other attention modules: it can enhance feature interactions in the global dimension while reducing information loss. GAM is derived from the Convolutional Block Attention Module (CBAM), retaining the sequential mechanism of channel-spatial attention while improving the subnetwork structure: the channel attention submodule adopts a three-dimensional arrangement to preserve complete dimensional information and combines a two-layer MLP to enhance cross-dimensional channel-spatial correlation; the spatial attention submodule fuses spatial information through two-layer convolution and focuses on extracting spatial features of pest regions.

Specifically, the upper input feature map F_1_ (Equation (1)) (∈R^C × H × W^) is first processed by the channel attention Mc, and F_2_ (Equation (2)) is obtained by elemental multiplication; F_2_ is then processed by the spatial attention Ms, and element-wise multiplication is performed again to generate F_3_ (Equation (3)). This channel-spatial sequential attention structure can not only preserve three-dimensional information but also amplify cross-dimensional feature dependencies, thereby more accurately capturing the subtle features of pests and effectively addressing the challenges of complex scenarios in pest detection.

F_1_ ∈ R^C × H × w^ (1)

F_2_ = M_C_(F_1_) ⊗ F_1_ (2)

F_3_ = M_C_(F_2_) ⊗ F_2_ (3)

### 2.4. EfficientNetv2 Lightweight Network

**Figure 5:**
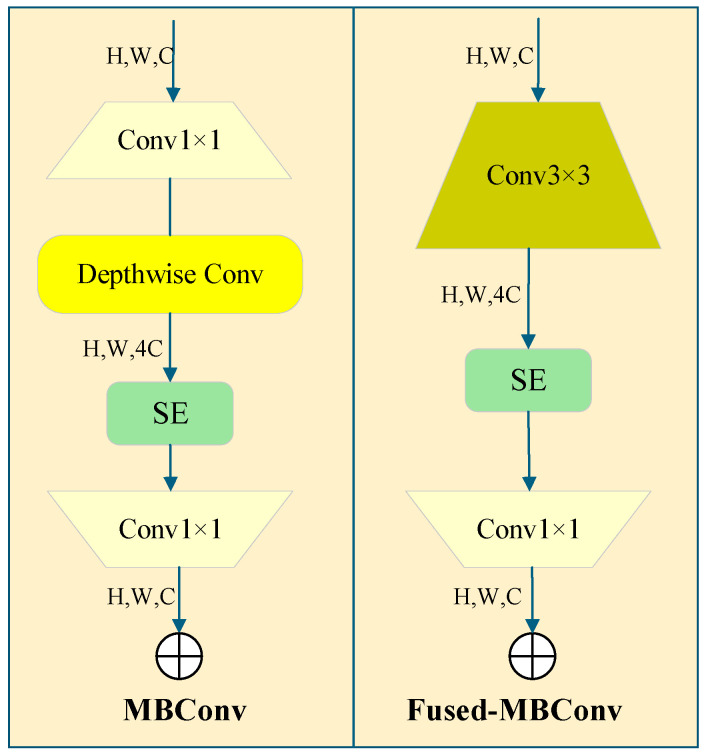
Schematic diagram of the MBConv.

To address the deficiencies of YOLOv8 in training efficiency, scale adaptability, and feature extraction, improvements can be made by introducing the core design of EfficientNetV2 [26]: The MBConv module was used to replace the traditional bottleneck module, which did not prioritize lightweight design. Through the split design of depth-wise separable convolution, cross-channel interference was avoided, and the fine-grained spatial features of small targets (such as contour pixels and local textures) were fully preserved. For the shallow layer of the backbone (the original feature generation layer for small targets), Fusded-MBConv was used to reduce information transmission loss caused by convolution splitting and to maximize retention of the original position and shape information of small targets. At the same time, the residual connection structure is retained, and the number of channels is flexibly adjusted via pointwise convolution to prevent the weak features of small targets from being diluted in the deep network. With the SE attention mechanism, weak target features are highlighted from a complex background through adaptive channel-weight learning, thereby greatly enhancing feature expression while balancing feature extraction quality and computational efficiency. By using the progressive training strategy and leveraging the efficient computational characteristics of the MBConv module, the image size was gradually increased over training rounds (320 → 480 → 640), allowing the small target to provide more pixel information. With adaptive adjustment of the regularization strength, the network efficiently converged while effectively reducing the risk of overfitting and achieved full learning of multi-scale features for small objects. The channel compression ratio of the neck feature fusion stage was optimized to reduce the information loss of small target features extracted by the pre-order module in the fusion process, and the integrity and effectiveness of feature fusion were improved. The specific network structure is shown in Figure 5.

### 2.5. Environmental Configuration and Parameter Settings

This experiment was conducted on Ubuntu 20.04.6 LTS with an NVIDIA A40 48 GB GPU. The software and hardware configurations were as follows: Python 3.11, CUDA 12.1, PyTorch 2.1.0, and Torchvision 0.16.0. During training, the batch size was 32, the number of training epochs was 250, the learning rate was 0.01, the weight decay coefficient was 0.0005, the optimizer was SGD, and the image size was 640 × 640 pixels.

### 2.6. Evaluation Index

This study adopted Precision (P), Recall (R), Mean Average Precision (mAP), and Parameter Count (Params) as the performance evaluation metrics of the model [27]. Among them, the calculation method of mAP is as follows: first, obtain the average precision of each pest category, then calculate the comprehensive mean of the average precision across all 9 pest categories. The mAP referred to in this study is usually mAP_0.5_, i.e., the mean average precision when the Intersection over Union (IoU) threshold is set to 0.5. The calculation methods of P, R, mAP_0.5_, and mAP_0.5–0.95_ are shown in Equations (4), (5), (6), and (7), respectively.

(4) $$P=\frac{\mathrm{TP}}{\mathrm{TP}+\mathrm{FP}}$$

(5) $$R=\frac{\mathrm{TP}}{\mathrm{TP}+\mathrm{FN}}$$

(6) $${\mathrm{mAP}}_{0.5}=\frac{\sum_{i=1}^{C}AP_{i}}{C}$$

(7) $$\mathrm{mAP}@0.5:0.95=\frac{\sum_{t=0.5}^{0.95}\mathrm{mAP}@t}{10}$$

Among them, TP refers to true positive samples, i.e., the number of correctly identified pests where the prediction box and the ground truth box belong to the same category and the IoU is greater than 0.5; FP refers to false positive samples, i.e., the number of samples incorrectly identified as pests; FN refers to false negative samples, i.e., the number of pests that are not identified; and C denotes the number of detection categories, where C was set to 9.

## 3. Results and Discussions

### 3.1. Performance Between FusedGM-YOLOv8 and Other Models

To evaluate the advantages of the proposed algorithm over current mainstream object detection models, this study conducted comparative experiments between the improved FusedGM-YOLOv8 model and Faster R-CNN, YOLOv5s, YOLOv8s, YOLOv10s, and YOLOv11s under the same training environments and parameter configurations. The results are shown in Table 2. It is evident that the improved model exhibited superior performance in terms of P, R, mAP_0.5_, mAP_0.5–0.95_, and parameter. Compared with the benchmark model YOLOv8s, the mAP_0.5_ and mAP_0.5–0.95_ of FusedGM-YOLOv8 have increased by 0.6% and 0.8%, respectively, while the parameter count has decreased by approximately 8%. These results demonstrate that the proposed model improves the lightweight level while maintaining detection accuracy. All performance metrics (P, R, mAP_0.5_, mAP_0.5–0.95_) of Faster R-CNN were much lower than those of the model proposed in this study. YOLOv5s had a slightly lower P than the model proposed in this study, while its Recall R, mAP_0.5_, and mAP_0.5–0.95_ were all lower than those of the proposed model, resulting in relatively low prediction accuracy. YOLOv10s exhibited a slightly higher P than the proposed model, but its R, mAP_0.5_, and mAP_0.5–0.95_ were slightly lower. Although YOLOv11s featured a relatively low parameter count, its P, R, mAP_0.5_, and mAP_0.95_ were all lower than those of the proposed model. Overall, the FusedGM-YOLOv8 model proposed in this study, while maintaining a relatively low parameter count, achieved performance on par with YOLOv8s in terms of the R metric. It also achieved a certain degree of improvement in other performance metrics (mAP_0.5_ and mAP_0.5–0.95_), with mAP_0.5–0.95_ in particular being significantly higher than that of the comparison models. This indicates that the model exhibited stronger detection capability for targets of different scales and effectively alleviated the occurrence of missed detections. Compared with existing studies, Huang Shirui [28] proposed a crop pest identification model based on the improved YOLOv7. The results showed that the model achieved an average accuracy of 80.4%, a precision of 85.3%, and a recall of 75.1%. It can be seen that its accuracy is lower than that of the proposed model in this study. Guo Jiaxuan [29] proposed a YOLOv5 crop pest identification model incorporating a global correspondence attention mechanism. The results showed that the model’s mAP_0.5_ and mAP_0.5–0.95_ were 72.3% and 47.0%, respectively, indicating that its accuracy is lower than that of the proposed model in this study. In summary, the model proposed in this study outperforms existing mainstream algorithms in comprehensive performance, combining the advantages of high accuracy and lightweight design, and thus has broad application prospects.

### 3.2. Performance of YOLOv8 Models Based on Different Attention Mechanisms

To assess the effectiveness of the proposed attention mechanism improvement algorithm, this study conducted comparative experiments on YOLOv8 models with different attention mechanisms (including YOLOv8s, YOLOv8_CA [30], YOLOv8_ECA [31], YOLOv8_CBAM [32], YOLOv8_SE [33], and YOLOv8_GAM) under the same training environment and parameter settings. The results are shown in Table 3. It is evident that compared with the base model YOLOv8s (P = 85.6%, R = 86.0%, mAP_0.5_ = 89.2%, mAP_0.5–0.95_ = 58.7%, parameter count = 11.1 M), all other models exhibited varying degrees of improvement across key performance metrics. Among these models, YOLOv8_GAM achieved a P of 85.7%, an R of 85.9%, an mAP_0.5_ of 89.7%, and an mAP_0.5–0.95_ of 59.1%. It performed the best in terms of mAP_0.5–0.95_ and comprehensive accuracy, which confirms its advantages in improving detection accuracy and multi-scale target recognition. YOLOv8_ECA and YOLOv8_CBAM both achieved an R of 86.3%, which was slightly higher than that of the base model, but their mAP_0.5–0.95_ values were slightly lower than those of YOLOv8_GAM. YOLOv8_SE achieved an R of 86.4%, and its comprehensive performance was slightly better than YOLOv8s but lower than YOLOv8_GAM. The performance of YOLOv8_CA was found to be comparable to that of the base model. Overall, the improved model proposed in this study achieved an overall improvement in P, R, and mAP metrics while maintaining an almost unchanged parameter count. Notably, it exhibits distinct advantages in terms of overall detection accuracy and multi-scale target detection capabilities.

### 3.3. Ablation Experiments

To verify the effectiveness of each proposed improvement for the pest detection task, this study used the original YOLOv8s model as the baseline and designed ablation experiments across different combinations of improved modules. The experimental results are shown in Table 4. As can be seen from Table 4, the baseline model without the GAM and MB modules achieved a P of 85.6%, an R of 86.0%, an mAP_0.5_ of 89.2%, an mAP_0.5–0.95_ of 58.7%, and a parameter count of 11.1 M. When only the MB module was added, the model’s parameter count decreased to 10.3 M, and the core metrics (P = 85.9%, R = 86.0%, mAP_0.5_ = 89.8%) exhibited relatively minor changes. The lightweight design of fusion convolution (1 × 1 point-wise convolution and 3 × 3 deep convolution fusion), grouped convolution, and residual connections, integrated by the MBConv module, improves the parallelism of feature extraction while simplifying redundant calculations and invalid parameter usage, and the residual connection effectively mitigates feature attenuation in deep networks. The SE mechanism employs a two-stage “compression-excitation” process. Firstly, the channel difference between the characteristics of pest targets (especially small targets) and background interference is perceived by global average pooling, and then the weight of fine-grained feature channels, such as larval contours, is increased by adaptive weight allocation, while suppressing invalid background interference, so that the limited parameters are focused on the core feature learning. This indicates that using the MB module alone mainly serves to reduce the parameter count and computational load, with limited improvement in detection accuracy. When only the GAM attention mechanism was added, the model’s parameter count increased to 11.2 M, while the P and mAP series metrics (P = 85.7%, R = 85.9%, mAP_0.5_ = 89.7%, mAP_0.5–0.95_ = 59.1%, Inference speed = 208.3 Frames per second) all improved. This indicates that the GAM can enhance the target feature extraction capability, improve the bounding box localization accuracy, and boost the overall detection performance. When both the GAM and MB modules were added, the model achieved optimal detection performance (P = 86.3%, R = 86.0%, mAP_0.5_ = 89.8%, mAP_0.5–0.95_ = 59.5%, Inference speed = 249.76 Frames per seconds), while its parameter count decreased to 10.2 M. This indicates a synergistic effect between the two modules: the MB module lays the foundation for lightweight design and efficient computation, and the GAM realizes feature enhancement and accuracy improvement, ultimately enabling the improved model to be both efficient and accurate in pest detection tasks.

### 3.4. Performance of Models Under Different Detection Scenarios

**Figure 6:**
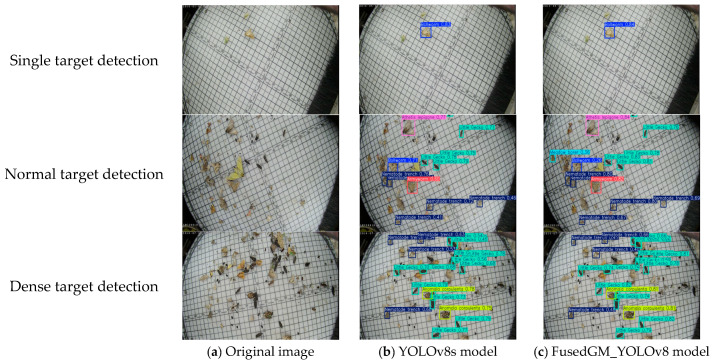
Model performance in different scenarios.

To further analyze the detection performance of the model proposed in this study across different detection scenarios, this subsection presents the detection results for pest images under three conditions: single target, normal target, and dense target, as shown in Figure 6. It can be seen that in the single-target scenario, the detection performance of the two models is similar. In the normal-target scenario, the advantages of FusedGM-YOLOv8 begin to become prominent: in most cases, it not only achieves higher recognition accuracy but also exhibits more efficient bounding box regression performance, with more precise localization and determination of pest targets. In the dense-target scenario, the performance advantages of FusedGM-YOLOv8 were further highlighted. It can successfully detect individual pests occluded in the center of the image, indicating that the model can more accurately focus on pest regions and effectively avoid occlusion interference. Based on the detection performance comparison results across these three scenarios, it can be seen that the FusedGM-YOLOv8 model outperformed the YOLOv8s model. It performed better in most cases, especially in bounding box regression efficiency, indicating that it is more suitable for the detection requirements of crop pests with different complexity levels.

### 3.5. Visual Analysis of Model Features

**Figure 7:**
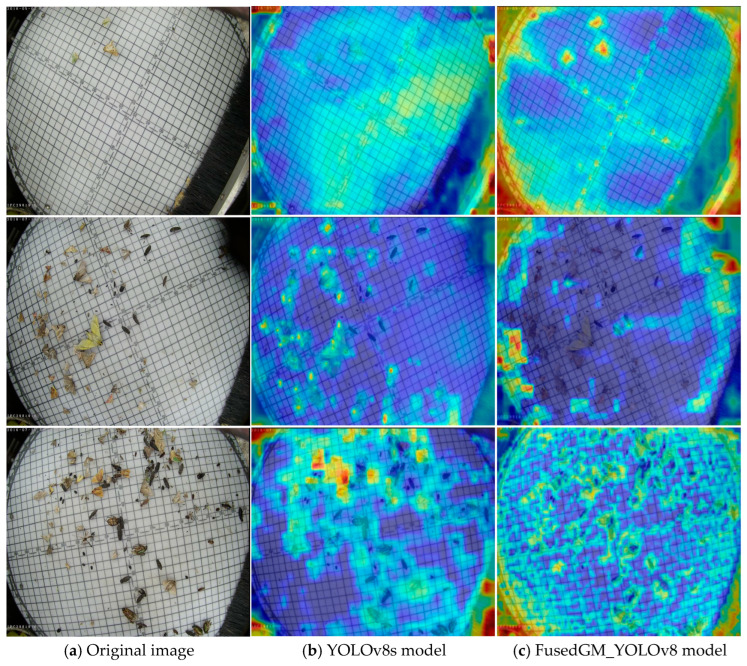
Visualization of feature map (The brighter the color (more towards red/yellow), the stronger the response intensity).

To visually validate the improvement in the FusedGM-YOLOv8 model in recognizing crop pests and diseases, this study adopted Grad-CAM [34] technology and visualized the model’s learning process of pest and disease features by generating heatmaps. The technical principle of Grad-CAM is as follows: relying on the backpropagation of training weights, the gradient matrix is first subjected to global average pooling in the spatial dimension, and then each channel of the feature layer is processed with weighted activation. The final heatmap clearly marks the feature regions in the image that have a key impact on the model’s prediction results based on the depth of regional brightness. By comparing the heatmap results of YOLOv8s and FusedGM-YOLOv8 (Figure 7), it can be seen that in the crop pest and disease target regions, the heatmaps generated by FusedGM-YOLOv8 not only exhibited brighter colors but also demonstrated significantly higher response intensities. This difference clearly demonstrated that FusedGM-YOLOv8 possessed a stronger ability to detect the correct pest and disease targets. It can accurately avoid interfering information, such as crop leaf textures and soil backgrounds, and focus on the key features of pest and disease regions (pest body details and disease spot edges). At the core of feature learning, this result further confirms the outstanding advantages of the model in the crop pest and disease recognition task.

**Figure 8:**
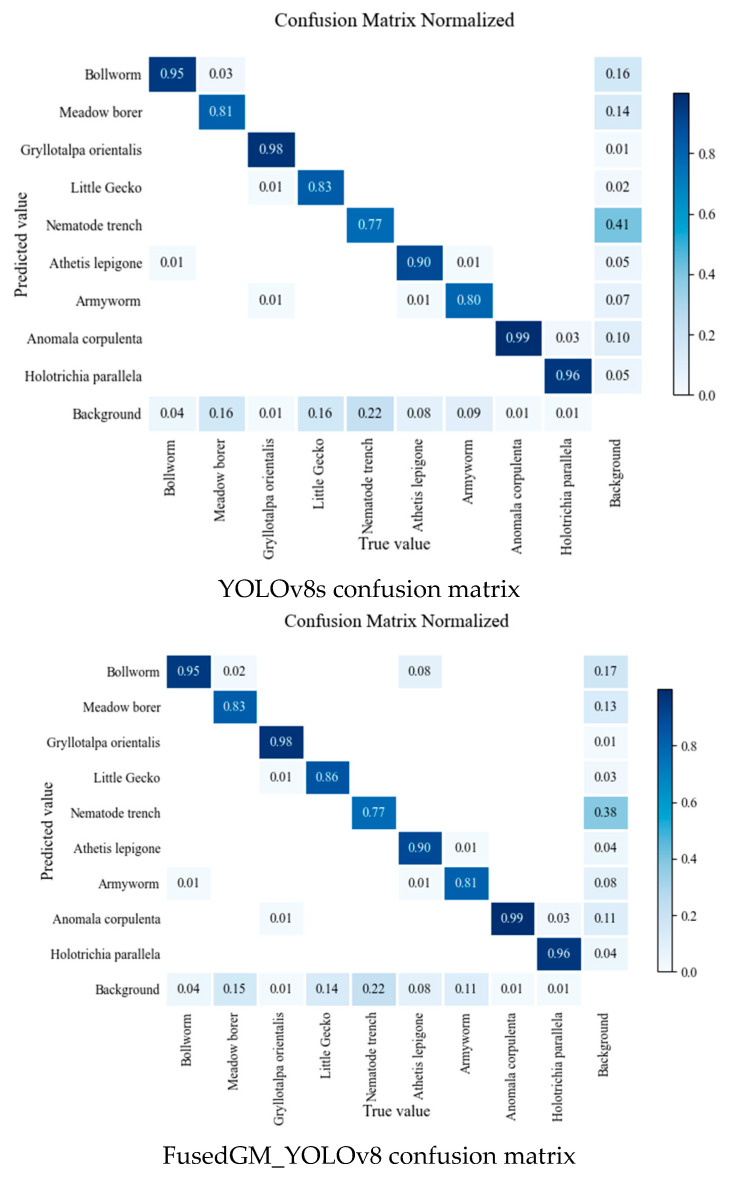
Confusion matrix of YOLOv8s and FusedGM_YOLOv8 model.

To compare the recognition performance among different pests, this study presents a comparison of the confusion matrices of YOLOv8s and FusedGM-YOLOv8, with the results summarized in Figure 8. It is evident that compared with YOLOv8s, FusedGM-YOLOv8 achieved better recognition performance for most pests. Specifically, FusedGM-YOLOv8 had higher recognition accuracy for small-target pests and pests with small sample sizes than YOLOv8s, indicating that it can more accurately recognize smaller pests and mitigate the impact of sample imbalance.

### 3.6. Experimental Validation

The improved FusedGM-YOLOv8 model was evaluated on the IP102 dataset. The experiment employed a Train from Scratch approach, with model parameters randomly initialized, and the IP102 dataset, which contains 96 pest types in natural scenes with complex occlusions, was used exclusively for training and evaluation. SGD served as the optimizer, with an initial learning rate of 0.01 and a cosine annealing schedule. The batch size was 32, and training continued for 300 iterations, with early stopping if the validation set loss failed to decrease for 15 consecutive rounds. The improved model achieved an mAP on the IP102 test set that was 2.7% higher than the original YOLOv8 (Table 5). These results indicate that the proposed strategies, including MBConv module replacement and progressive training, effectively address the complex data distribution of the IP102 dataset and improve model performance in pest detection tasks in natural scenes.

### 3.7. Design of the Real-Time Monitoring System

**Figure 9:**
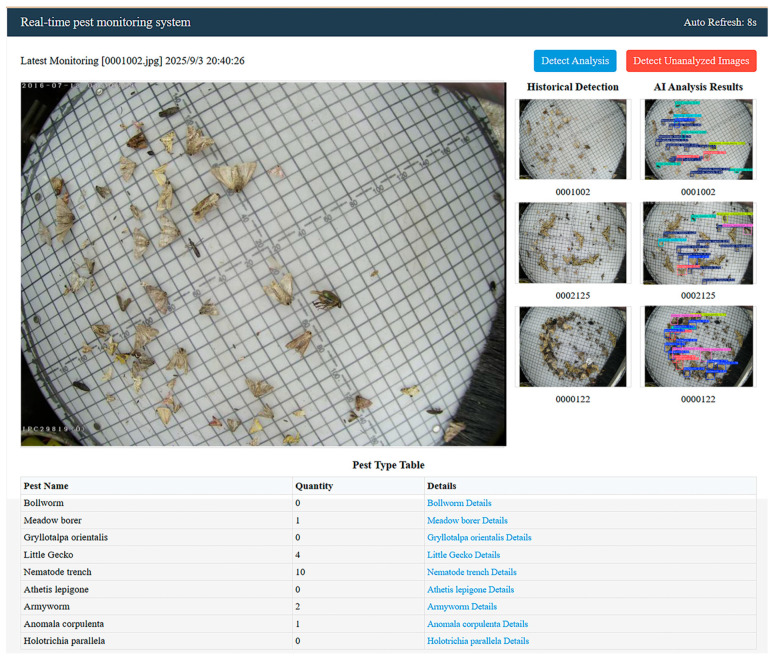
Real-time pest monitoring system.

Based on the improved YOLOv8s model, this study develops a real-time monitoring system for agricultural pest monitoring and constructs a full-closed-loop workflow of “edge collection—cloud reasoning—front-end visualization”. The system hardware integrated a high-definition camera, a fill light, and an edge device box, and supported dual-mode timing/real-time acquisition. Images were compressed, locally cached, and uploaded to the Alibaba Cloud server via a 4G/Wi-Fi dual-mode network (including a breakpoint for continued transmission). The B/S architecture front-end is deployed in the cloud, supporting functions such as double image comparison between original images and labeled images, pest species/number, and Baidu Encyclopedia knowledge link query. Users can obtain visual results by triggering analysis with one click (Figure 9). In the face of complex situations in farmland, the average time of end-to-end (manual trigger analysis on demand) of the system was ≤3.0 s, ≤5.5 s and ≤7.8 s, respectively, under three different network states: high-quality network, conventional network and weak network, meeting the needs of real-time monitoring. To address the pain point of network instability in agricultural environments, a dynamic compression, fragment transmission, and local cache adaptation mechanism is designed. Images can be cached for 30 days when the network is disconnected, automatically transmitted after recovery, and run continuously for 72 h without crashing.

## 4. Conclusions

This study takes nine different types of crop pests as research objects, and an improved lightweight pest identification model FusedGM-YOLOv8, was proposed to address the limitations of the existing YOLOv8 model in terms of detection accuracy and deployment adaptability. Based on this improved model, a real-time pest monitoring system with an “end-edge-cloud” architecture was further developed. The main conclusions are as follows:

1. A dataset containing nine categories of crop pests was constructed, with a total of 7101 images, providing a pest dataset targeting pest states for subsequent research.
2. Based on the YOLOv8 model, a lightweight attention mechanism and a feature enhancement module were incorporated. The results showed that under the same experimental conditions, the FusedGM-YOLOv8 model exhibited better detection performance on the self-constructed pest dataset than the Faster R-CNN, YOLOv5s, YOLOv8s, YOLOv10s and YOLOv11s models. Compared with the original YOLOv8s model, its mAP_0.5_ and mAP_0.5–0.99_ increased by 0.6% and 0.8%, respectively. Compared with YOLOv8 models integrated with other attention mechanisms, the improved model proposed in this study achieved overall improvements in P, R, and mAP metrics while maintaining nearly identical parameter counts and exhibited significant advantages, especially in terms of comprehensive accuracy and multi-scale target detection capability.
3. Under three typical scenarios (single target, normal target, and dense target), the FusedGM-YOLOv8 model exhibited better pest detection performance than the original YOLOv8s model. It can not only accurately identify smaller-sized pest individuals but also improve the recognition accuracy of various pests, fully demonstrating the optimization effect of the improved strategy on detection performance. Feature map visualization results indicated that the FusedGM-YOLOv8 model possessed a stronger ability to perceive the correct pest and disease targets and could focus on the key features of pest and disease regions.
4. The FusedGM-YOLOv8 model also exhibited significant advantages on the IP102 dataset with unbalanced data volume distribution. Compared with the original YOLOv8s model, its P, mAP_0.5_, and mAP_0.5–0.95_ increased by 2.6%, 2.7%, and 1.4%, respectively.

The current improved algorithm still has room for improvement. The datasets used in existing experiments cover a limited number of pest species, which may affect the model’s adaptability to diverse field pests. Future research will focus on optimizing the model architecture to improve reasoning speed and investigate a hybrid approach that integrates deep learning with manual rules. Deep learning methods are used for feature extraction, while manual rules address the identification challenges posed by rare pest species. Also, further supplement more types and quantities of pest and disease sample data, optimize the model structure to enhance inference speed, and continuously improve the algorithm to further increase detection accuracy, thereby providing more powerful technical support for the deployment of the model on edge devices and its practical field applications.

## Figures and Tables

**Table 1:** Number of each type of pest.

Pest Types	Training Set	Validation Set	Test Set
Bollworm	4401	595	538
Meadow borer	3341	432	390
Gryllotalpa orientalis	3138	360	467
Little Gecko	1395	169	120
Nematode trench	1932	313	247
Athetis lepigone	890	138	135
Armyworm	1093	168	122
Anomala corpulenta	4529	578	614
Holotrichia parallela	853	115	142

**Table 2:** Performance of different models on the test set.

Model	P/%	R/%	mAP_0.5_/%	mAP_0.5–0.95_/%	Parameters/×10^6^ M
FasterR-CNN	43.1	58.0	47.7	37.5	41.4
YOLOv5s	86.2	84.6	88.1	55.7	7.2
YOLOv8s	85.6	86.0	89.2	58.7	11.1
YOLOv10s	86.5	84.9	89.4	58.2	7.2
YOLOv11s	85.0	84.5	89.2	58.6	9.5
FusedGM_YOLOv8	86.3	86.0	89.8	59.5	10.2

**Table 3:** Performance of YOLOv8 model based on different attention mechanisms.

Model	P/%	R/%	mAP_0.5_/%	mAP_0.5–0.95_/%	Parameters/×10^6^ M
YOLOv8s	85.6	86.0	89.2	58.7	11.1
YOLOv8_CA	85.3	86.2	89.0	58.5	11.2
YOLOv8_ECA	85.3	86.3	89.1	58.7	11.5
YOLOv8_CBAM	85.1	86.3	89.3	58.6	11.8
YOLOv8_SE	85.1	86.4	89.1	58.6	11.2
YOLOv8_GAM	85.7	85.9	89.7	59.1	11.2

**Table 4:** Results of ablation test (Note: “√” indicates the adoption of the corresponding strategy, “×” indicates the nonadoption of the corresponding strategy).

GAM	MB	P/%	R/%	mAP_0.5_/%	mAP_0.5–0.95_/%	Parameters/×10^6^ M	Inference Speed/Frames Per Seconds
×	×	85.6	86.0	89.2	58.7	11.1	225.38
×	√	85.9	86.0	89.8	59.2	10.3	286.64
√	×	85.7	85.9	89.7	59.1	11.2	208.30
√	√	86.3	86.0	89.8	59.5	10.2	249.76

**Table 5:** Performance of the model on the IP102 dataset.

Model	P/%	R/%	mAP_0.5_/%	mAP_0.5–0.95_/%
YOLOv8s	55.5	59.8	57.6	37.7
FusedGM_YOLOv8	58.1	56.6	60.3	39.1

## Data Availability

The data presented in this study are available on request from the corresponding author.
